# Difficulties associated with the interpretation of postmortem toxicology

**DOI:** 10.1093/jat/bkae052

**Published:** 2024-06-08

**Authors:** Lilli Stephenson, Corinna Van Den Heuvel, Timothy Scott, Roger W Byard

**Affiliations:** School of Biomedicine, The University of Adelaide, Adelaide, SA 5005, Australia; School of Biomedicine, The University of Adelaide, Adelaide, SA 5005, Australia; Forensic Science SA (FSSA), Adelaide, SA 5000, Australia; College of Science and Engineering, Flinders University, Bedford Park, SA 5042, Australia; School of Biomedicine, The University of Adelaide, Adelaide, SA 5005, Australia; Forensic Science SA (FSSA), Adelaide, SA 5000, Australia

## Abstract

While postmortem (PM) toxicology results provide valuable information towards ascertaining both the cause and manner of death in coronial cases, there are also significant difficulties associated with the interpretation of PM drug levels. Such difficulties are influenced by several pharmacokinetic and pharmacodynamic factors including PM redistribution, diffusion, site-to-site variability in drug levels, different drug properties and metabolism, bacterial activity, genetic polymorphisms, tolerance, resuscitation efforts, underlying conditions, and the toxicity profile of cases (i.e. single- or mixed-drug toxicity). A large body of research has been dedicated for better understanding and even quantifying the influence of these factors on PM drug levels. For example, several investigative matrices have been developed as potential indicators of PM redistribution, but they have limited practical value. Reference tables of clinically relevant therapeutic, toxic, and potentially fatal drug concentrations have also been compiled, but these unfortunately do not provide reliable reference values for PM toxicology. More recent research has focused on developing databases of peripheral PM drug levels for a variety of case-types to increase transferability to real-life cases and improve interpretations. Changes to drug levels after death are inevitable and unavoidable. As such, guidelines and practices will continue to evolve as we further our understanding of such phenomena.

*All things are poisons, for there is nothing without poisonous qualities. It is only the dose which makes a thing poison*.

Paracelsus (1493–1541)

## Introduction

Postmortem (PM) toxicology is a common and important ancillary analysis informing coronial investigations which may assist in the determination of both cause and manner of death. However, difficulties arise in the interpretation of PM drug levels. While concentrations of a drug in blood in a clinical setting can be used to provide an estimate of the dose that was administered, several (often unknown) factors impact the reliability of such analyses including dose timing and rate of metabolism. In a PM setting, difficulties in toxicological interpretation are further confounded by several physiological and environmental variables which may change PM drug levels. After death, a series of changes ensue that collectively influence the distribution of compounds in the body over time. As such, a PM drug concentration may not be an accurate representation of the drug concentration that was present during life [1]. For this reason, dose estimations should only be made with an acknowledgement of the limitations of interpreting PM results [2, 3].

## Postmortem redistribution

Postmortem redistribution (PMR) is a term that describes the time- and site-dependent changes to drug levels that occur after death that are influenced by a number of physiological and pharmacokinetic factors [4–8]. Research on this phenomenon began in the 1960s as a result of temporal changes that had been identified between central and peripheral PM barbiturate blood levels [9, 10]. Since that time, a large body of research has been dedicated to gaining a better understanding of the factors that influence PMR for a wide range of drugs, including barbiturates, antipsychotics, benzodiazepines, and opioids. Factors influencing PMR include PM changes (e.g. putrefaction, decomposition), the pharmacokinetic properties of the drug, and anatomical variations in tissues.

## Postmortem diffusion

A significant factor in PMR involves passive diffusion of compounds across a concentration gradient, from an area of high concentration (e.g. stomach, liver, lung) to low concentration (e.g. blood) after death [11–14]. In the absence of oxygen and an inability to produce adenosine triphosphate (ATP), the energy-dependent systems that are integral in maintaining concentration gradients across cell membranes are lost. Loss of membrane integrity causes the release of intracellular enzymes and subsequent cell lysis (autolysis). Furthermore, the release of other compounds associated with anaerobic metabolism, including lactic acid and formic acid, causes a decrease in pH levels [15, 16]. In this newly established anaerobic environment, these changes cause further degradation of cells and tissues and collectively contribute to the PM processes of putrefaction and decomposition [17]. Changes to drug–tissue binding occur in parallel to these PM processes, allowing for the passive diffusion of drugs throughout the body [7, 18]. Depending on the pharmacokinetic properties of drugs, they may either diffuse from the tissues back into the blood or into surrounding organs and tissues.

## Drug reservoirs and site-to-site variability of drug concentrations

While cell membrane integrity, channel selectivity, and drug–tissue binding decline after death, the diffusion of drugs is not uniform. For example, up to seven-fold differences in blood drug concentrations have been observed between different sample sites in a body [19–21]. However, this has been shown to vary greatly between different compounds which is attributed to PMR and site-specific accumulation of drug for particular compounds such as digoxin in cardiac muscle or tricyclic antidepressants in the liver [18, 22]. Differences of up to 760% for imipramine have been observed between pulmonary and peripheral venous blood samples [18]. The tissue-binding characteristics of a compound will determine where the most profound effects of PMR are observed. For example, a compound that accumulates in the liver will readily diffuse into the hepatic venous blood and surrounding structures by following concentration gradients [18].

Organs that are integral to the binding and/or metabolism of drugs are referred to as ‘drug reservoirs’, and include the gastrointestinal tract, lungs, myocardium, and liver [5]. Drugs present within these tissues will redistribute throughout the body after death by diffusing into surrounding tissues, blood, or other bodily fluids with demonstrable site-dependence. One example of this phenomenon is the diffusion of compounds (e.g. ethanol) from the stomach contents into the surrounding tissues [13, 14, 23–30]. The extent of PMR from the stomach may also be influenced by the volume of stomach contents and the amount of drug ingested or administered [14]. However, the most well studied example of this phenomenon involves diffusion of compounds from the heart into cardiac blood, resulting in artefactually raised PM cardiac blood levels compared to levels in the peripheral blood [31, 32]. As such, the extent of PMR is most commonly evaluated by calculating the cardiac to peripheral (femoral) blood concentration ratio [31]. There are additional factors that may cause variations to this ratio including age, sex, dose, and drug pharmacokinetics [33].

The route of administration is also a factor affecting the extent and location of PM diffusion. PM gastroesophageal reflux has also been shown to cause drugs to diffuse from the oesophagus into the aorta, resulting in artefactually elevated aortic blood concentrations [27]. However, aortic blood is rarely utilized in routine forensic toxicological analyses, so this is not practically relevant. In general, the extent of PMR is thought to be influenced by the distance from the site of the drug and the concentration (i.e. the further the distance, the lower the capacity for redistribution) [11, 12, 14]. Basic drugs present in high concentrations in the lungs will diffuse rapidly into the cardiac chambers via the pulmonary venous blood rather than by concentration gradients. However, in the early PM period, this diffusion may not greatly contribute to increases in cardiac blood drug concentrations, although it may be dependent on movement of the body after death [34, 35].

## Drug properties

Drug properties that have been shown to influence pharmacokinetics and PMR include lipophilicity, the degree of ionization (pKa), and volume of distribution (Vd). Lipophilicity is a term which describes the tendency of a drug to dissolve in lipids (fat) and pKa describes how strong or weak an acid is. Most drugs are either weak acids or bases, and therefore exist in both ionized and non-ionized forms with the ratio dictated by intracellular pH [36]. During life, the pH of blood and stomach contents are approximately 7.4 and 3 (ranging from 2 with fasting and 5 after food consumption), respectively [37, 38]. However, after death, cell lysis will cause the physiological pH to become increasingly acidic (up to a value of 5.5) [6, 15]. Therefore, lipophilic, basic drugs that accumulate in organ tissues (e.g. lungs, liver and myocardium) during life will become more ionized, allowing for passive diffusion of the drug after death and more profound PMR [6, 7].

Vd is the amount of drug present within the body divided by the plasma concentration [6]; lipophilic drugs that distribute into muscle, adipose tissue, and other intracellular components have a high Vd [39]. Conversely, drugs that bind to plasma proteins but not tissues have a lower Vd [6]. A drug is considered more likely to undergo PMR if the Vd is greater than 3–4 L/kg [11, 40]. Compounds with basic, lipophilic characteristics, such as certain antipsychotic drugs, undergo marked redistribution into peripheral body cavities after death [6, 41–44]. A study of PMR which compared the concentration of antipsychotic compounds in the femoral blood at the time of mortuary admission and then at autopsy showed significant changes in drug concentration of up to 40% in either direction [42]. Such compounds are highly tissue-bound and may therefore diffuse from tissues back into the blood after death [22, 45, 46]. However, the opposite effect may also occur if there has not been sufficient time for equilibrium to be achieved between blood and tissues, for example in cases of acute drug toxicity [44].

## Drug metabolism

After death, there is also a gradual reduction in enzyme activity, although the activity of drug-metabolizing enzymes may continue for the first few hours after death. A study of CYP450 enzymes in rats showed less than 50% enzyme activity 24 hours PM and a 90% reduction by 48 hours [47]. PM enzyme activity also contributes to changes in the concentrations of drugs after death. For example, PM conversion of amitriptyline to nortriptyline was especially pronounced in lung tissue where enzymes responsible for metabolizing the drug are more concentrated [23]. PM hydrolysis of morphine can result in more apparent ‘free’ (unconjugated) morphine (as opposed to total morphine which includes drug released after hydrolysis of conjugated metabolites) [48].

Differences in the half-life of drugs can introduce difficulties in assessing drug use during life. For example, morphine which has a half-life of approximately 1–3 hours is likely to be detected in PM toxicology if the decedent had used morphine or heroin. However, the principle metabolite of heroin, 6-monoacetylmorphine (6-MAM), which has a half-life of approximately 30 minutes, is often degraded in the time between administration and death, before PM blood samples are taken. As such, 6-MAM may not be detected in the PM blood in a heroin-related death [49, 50]. In the absence of 6-MAM, it is much more difficult to differentiate between the use of heroin and other morphine or codeine-containing products. Evaluation of a variety of biomarkers provides the most reliable indication of heroin use (e.g. 6-MAM, codeine, heroin impurities, etc.) [51].

Drug interactions may complicate the interpretation of PM levels, particularly if several drugs are metabolized by the same enzyme, resulting in the accumulation of drugs with lower binding affinity [52].

## Bacterial activity

During life, the body is generally sterile apart from the gastrointestinal tract and cavities such as the mouth and vagina, or when sepsis is present. Bacteria present after death may contribute either to the production or degradation of compounds in addition to accelerating the PM processes of putrefaction and decomposition [53].

For example, ethanol may be produced after death as a result of microbial activity and the fermentation of glucose, which increases with the PM interval and degree of putrefaction [54]. For this reason, rapid body recovery and refrigeration are required to reduce PM ethanol production [55]. PM ethanol levels of 70 mg/dL or less generally occur due to bacterial activity but may be as high as 220 mg/dL in markedly decomposed bodies [44, 56]. A study which evaluated PM ethanol levels with consideration of antemortem alcohol consumption and the state of decomposition concluded that PM ethanol levels greater than 200 mg/dL are most likely not solely due to PM production [56]. In addition to taking a femoral vein blood sample, samples of urine and vitreous humor (VH) are useful for comparative purposes [57]. The evaluation of PM samples for other microbial ethanol products (e.g. n-propanol and n-butanol) may provide additional discriminatory evidence [58–60]. In general, ethanol studies in moderately or markedly decomposed bodies are more difficult to interpret due to the increased opportunity for bacterial ethanol production [28].

Gamma hydroxybutyric acid (GHB) is another compound that is produced endogenously [61], but is also a drug of abuse. When GHB emerged as a drug of abuse, the detection, quantification, and interpretation of PM levels became problematic. Historical and recent studies have had difficulty in differentiating between endogenous and drug-related GHB levels as it is often detected in both blood and urine [62]. It is now generally understood that PM GHB levels of less than 30 mg/L in blood and 20 mg/L in urine can be regarded as endogenous in origin, although low concentrations may also be present from prior use [63]. However, as for ethanol, much higher concentrations may also be detected in bodies with advanced decomposition.

While microorganisms may be involved in the PM production of certain compounds (e.g. ethanol), they may also be responsible for metabolism of others. In particular, PM bioconversion of nitrobenzodiazepines (e.g. flunitrazepam, clonazepam, and nitrazepam) by bacteria has been demonstrated in response to changes in pH and temperature associated with putrefaction [64]. Several studies of bacterial degradation of antipsychotic medications in decomposing, unpreserved blood samples have been conducted over the last two decades [53, 65, 66]. The presence of 2-hydroxybenzoyl-risperidone and 2-hydroxybenzoyl-paliperidone in samples where risperidone and paliperidone are detected indicates a decrease in the latter due to PM bacterial degradation [53]. A study of serotonin-selective antidepressants showed stability for venlafaxine, citalopram, fluoxetine, and paroxetine, but potential concerns were raised about bacterial degradation of fluvoxamine [66]. However, current sampling guidelines utilizing sodium fluoride largely inhibit such processes from occurring post-sampling but are obviously dependant on PMI as well as other environmental factors.

Bacterial degradation of drugs increases with increasing temperature as demonstrated for other compounds, particularly for internal temperatures between 20°C and 37°C [53]. Additional external factors that may accelerate decomposition and bacterial degradation include trauma to the body (such as in a motor vehicle accident), domestic heating, enclosed vehicles, blankets or tight clothing, and some medical conditions including diabetes mellitus, sepsis, and obesity [67].

## Other considerations

### Genetic polymorphisms

Even in controlled environments, PMR demonstrates a high degree of inter-individual variability [30]. This may partially be attributed to differences in individual drug metabolizing capabilities. For example, cytochrome P450 (CYP) enzymes demonstrate significant inter-individual variability which is attributed to genetic polymorphisms which may either increase or decrease enzyme activity resulting in subsequent variations in drug metabolism [52, 68]. Depending on the phenotype, individuals may be categorized into the following groups: poor metabolizers, intermediate metabolizers, extensive metabolizers, and ultra-rapid metabolizers [68]. For example, individuals with an ultra-rapid metabolizer phenotype may demonstrate high morphine/codeine ratios after consumption of only codeine [69]. Phenotypes have also demonstrated ethnic variability [68, 70]. For example, single nucleotide gene polymorphisms of several CYP enzymes showed that the CYP2C19*2 mutation resulted in poor metabolism of zolpidem in the Chinese Han population [68].

### Influence of tolerance

Tolerance, particularly in the setting of opioid use, may be a key consideration in the evaluation of potentially toxic blood concentrations. Tolerance may develop because of pharmacodynamic or pharmacokinetic adaptations after repeated systemic exposure resulting in a long-term decrease in drug response [71]. An example of loss of tolerance may occur with recently released prisoners who die taking a dose of heroin that was tolerable prior to incarceration, but was subsequently not due to lack of continued exposure [72].

### Resuscitation efforts

Cardiopulmonary resuscitation and movement of the body may also contribute to PMR through physical drug redistribution [6, 73, 74]. For this reason, blood samples taken as early as possible during resuscitative efforts may provide the most accurate assessment of levels prior to the terminal episode [74].

### Underlying conditions

Deaths involving drug use with underlying chronic disease, particularly those that cause reduced cardiorespiratory function [e.g. chronic obstructive pulmonary disease (COPD)], require detailed documentation of the decedent’s medical history for the most accurate determination of the specific roles and potential interactions of drug toxicity and organic illness. For example, the respiratory depressant effects of opioid drugs may act synergistically with underlying COPD, resulting in death. Fatal outcomes have also been associated with the effects of amphetamine use with pre-existing heart disease [75] and positional asphyxia in obese decedents in the setting of drug use [76].

### Mixed drug toxicity deaths

The use of several drugs, particularly those with additive respiratory depressant effects (e.g. opioids and benzodiazepines), may further complicate the interpretation of potentially toxic blood concentrations. For example, it has been shown that high levels of alcohol are associated with a greater risk of heroin (morphine) overdose [77–79]. Many common antidepressant and antipsychotic medications are also central nervous system depressants, producing increased toxicity when taken in combination with alcohol and/or opiates.

## Potential investigative matrices

### Central:peripheral blood concentration ratio

The ratio of central blood to peripheral blood (C/P ratio), also referred to as the cardiac to femoral blood ratio, has been proposed as a potential indicator of PMR. A ratio of greater than 1 is thought to indicate PMR, where higher ratios indicate greater PMR [4, 73, 80, 81]. However, rat models used to demonstrate the utility of this formula have had highly variable results [73]. Some drugs have demonstrated only minor changes (e.g. sertraline) [82, 83] while others have shown more marked alterations (e.g. venlafaxine, fluoxetine, fluvoxamine) [31, 83–86]. Several studies have shown that drug concentrations may actually ‘increase’ in peripheral blood relative to central blood, as is the case with tetrahydrocannabinol [87, 88]. Caution is therefore warranted when interpreting these ratios. Such differences may be attributed to the sample site, time of sampling, characteristics of the drug, or simply to differences in drug distribution during life [31, 81]. In general, the ratio of cardiac to peripheral drug concentrations will increase over time [8, 11, 86], although it may also decrease [89, 90]. Clinical studies have shown that peripheral drug concentrations may be lower due to incomplete absorption of a drug at the time of sampling [91]. Drugs may also not have sufficient time to reach a steady state, particularly with intravenous drug administration which will impact interpretation of this ratio [81, 92]. Alternatively, while a drug such as fentanyl may be taken orally, it may also be administered transdermally in the form of a patch. In cases involving transdermal administration of fentanyl with the suspicion of fatal intoxication, the C/P ratio has been used as supportive evidence of lethal drug toxicity [93, 94]. Other factors which may influence the rate of absorption in cases of transdermal administration include the type of patch, integrity of the skin, body temperature, and even the presence of cosmetic agents [95, 96]. It is also important to note that peripheral (femoral) blood may also show PMR, so it may not necessarily be a stable reference point for comparisons against cardiac blood levels. While femoral blood is the most commonly collected peripheral sample, significant variation in sampling site and technique (e.g. ligation, ‘blind stick’) between pathologists likely contributes to further ambiguity.

### Vitreous humor:femoral blood concentration ratio

The drug concentration ratio in VH and femoral blood (VH/FB ratio) has been used to predict survival time in heroin-related deaths, with a lower ratio expected in rapid deaths due to insufficient time for redistribution into the VH and vice versa [79]. Conversely, high ratios may be indicative of more extensive PMR into the VH, metabolism of morphine in the blood, and conversion of 6-MAM into morphine due to the relatively neutral pH of human VH [79, 97]. For many drugs however, little is known about their expected concentrations in VH, making interpretation of the VH/FB ratio difficult.

### Metabolite:parent drug ratio

Metabolite to parent drug concentration ratios have been used to estimate dose, where a low ratio may indicate acute drug toxicity [98, 99]. However, interpretation of these ratios is dependent on the PMR behaviours of each drug. For example, a study which compared the ratios between amitriptyline and venlafaxine (and their respective metabolites) reported the ratio of the former to be within normal range and the latter to be below normal even with moderate to high doses [99]. However, interpretation of this matrix also requires context. For example, a low ratio may be present in a decedent with a history of chronic use, where death was caused by an acute overdose. Conversely, a high ratio may be observed in a decedent with reduced metabolic function due to drug interactions or a poor metabolizer phenotype [52].

### Free:total drug concentration ratio

Studies of free (unconjugated) and total morphine concentration ratios have been used to estimate the survival period (rapid versus delayed) following morphine (heroin) administration [100–103]. A free:total ratio closer to 1 generally indicates a rapid death due to the reduced time for conversion of morphine to its conjugated forms [50, 79, 101, 104, 105]. Some studies have reported more specific ranges, between 0.51 and 0.7 for rapid deaths and between 0.31 and 0.34 for delayed deaths [101, 104, 105]. However, as with all measures, this application may also be confounded by the effects of PMR. Any conclusions drawn from free and total drug concentrations have to be treated with caution as cases with low ratios have also been associated with short survival times [79, 106]. Explanations for low ratios in rapid deaths include the accumulation of morphine glucuronides in habitual morphine/heroin users or renal insufficiency [104, 107]. Conversely, high ratios may also occur in delayed deaths due to PMR or hydrolysis of morphine glucuronides due to PM bacterial activity [29, 48, 106, 108]. Furthermore, variable ratios of morphine metabolites may also be observed if the drug has been administered intravenously, thereby not allowing first pass metabolism to occur [107].

The free:total ratio has also been applied to assist with differentiating heroin from morphine use [49]. Burt et al. found that cases with detectable 6-MAM demonstrated significantly higher free to total morphine ratios than those without [102]. However, it has been proposed that reporting free (unconjugated) morphine as opposed to total morphine concentrations provides a better indication of toxicity, due to the unequal production of the glucuronidated conjugates (the inactive 3-isomer and the active 6-isomer) captured in total morphine values [50]. Other potential markers of PMR have been investigated such as hepatic enzyme levels, but there is poor correlation [109].

### Endogenous compound markers

In recent years, there has been a focus on metabolomic studies to find endogenous compounds that may mimic the PM behaviour of certain drugs, thus allowing an assessment of any PMR that has occurred [110, 111]. This strategy has promise; however, no endogenous markers have yet been discovered which are robust enough to be applied to forensic cases.

## Reporting values

Reference tables for therapeutic, toxic, and potentially fatal drug concentrations are available for clinical applications [112–119] but may be potentially misleading in the context of PM investigations. As such, therapeutic plasma concentrations should not necessarily be used as reference intervals for PM toxicology [120]. Furthermore, such tables often give therapeutic and toxic concentrations in the absence of corresponding reference time intervals and whether a sample is from blood or plasma [120]. However, several studies have been undertaken in recent years which report PM concentration ranges between fatal and non-fatal cases, and single- and mixed-drug toxicity deaths [121–123]. A Swedish database of single- and mixed-drug toxicity cases with corresponding femoral PM drug levels provides a framework for an approach to creating a database of more reliable PM reference values [121]. However, issues also arise in evaluating the significance of drug levels in the very young as reference ranges are based on adult studies. Further work is required to address such limitations including documentation of pre-existing underlying disease for better transferability to real-life cases. While still in the early stages of development, statistically supported interpretative tools have been proposed which may provide more robust toxicological evaluations in cases where decedent and drug information is limited [124].

Further difficulties arise in assessing blood concentrations of new psychoactive substances (NPS) such as synthetic cannabinoids [125]. In some cases, reference samples and standards are not yet available which prevents quantification of PM drug levels. Where reference samples are available, the literature on fatal and non-fatal concentrations is limited, further impacting on the interpretation of such results.

Where compilations of drug concentrations are reported in the literature, it is recommended that values be reported as median values and percentiles rather than means with standard deviation to reduce the influence of extreme values on average concentrations [102].

## Conclusion

Changes to drug levels after death are inevitable and unavoidable. As such, guidelines and practices will continue to evolve as we further our understanding of such phenomena. While work has been done to create databases of reference concentrations with inclusion of various factors that may skew results, further study is still required to clarify the potential interaction of natural diseases and drugs detected PM, and to determine the influence of various PM changes on antemortem levels. However, despite the uncertainties associated with PM measurements, the information provided by PM toxicology remains invaluable in a wide range of cases, contributing to the determination of the cause, mechanism, and manner of death.

## Data Availability

No new data were generated or analyzed in support of this research.

## References

[1] Maskell PD . Just say no to postmortem drug dose calculations. *J Forensic Sci* 2021;66:1862–70. doi: 10.1111/1556-4029.14801 34302366

[2] Elliott SP, Stephen DWS, Paterson S. The United Kingdom and Ireland Association of Forensic Toxicologists forensic toxicology laboratory guidelines. *Sci Justice* 2018;58:335–45. doi: 10.1016/j.scijus.2018.05.004 30193659

[3] AAFS Standards Board . ANSI/ASB best practice recommendation 037: guidelines for opinions and testimony in forensic toxicology. 2019. https://www.aafs.org/sites/default/files/media/documents/037_BPR_e1.pdf (1 August 2023, date last accessed).

[4] Cook DS, Braithwaite RA, Hale KA. Estimating antemortem drug concentrations from postmortem blood samples: the influence of postmortem redistribution. *J Clin Pathol* 2000;53:282–85. doi: 10.1136/jcp.53.4.282 10823124 PMC1731178

[5] Pélissier-Alicot AL, Gaulier JM, Champsaur P et al. Mechanisms underlying postmortem redistribution of drugs: a review. *J Anal Toxicol* 2003;27:533–44. doi: 10.1093/jat/27.8.533 14670131

[6] Yarema MC, Becker CE. Key concepts in postmortem drug redistribution. *Clin Toxicol* 2005;43:235–41. doi: 10.1081/CLT-58950 16035199

[7] Ferner R . Postmortem clinical pharmacology. *Br J Clin Pharmacol* 2008;66:430–43. doi: 10.1111/j.1365-2125.2008.03231.x 18637886 PMC2561112

[8] Kennedy MC . Postmortem drug concentrations. *Internal Med J* 2010;40:183–87. doi: 10.1111/j.1445-5994.2009.02111.x 19849743

[9] Curry AS, Sunshine I. The liver:blood ratio in cases of barbiturate poisoning. *Toxicol Appl Pharmacol* 1960;2:602–06. doi: 10.1016/0041-008X(60)90026-0

[10] Parker JM, Winek CL, Shanor SP. Postmortem changes in tissue levels of sodium secobarbital. *Clin Toxicol* 1971;4:265–72. doi: 10.3109/15563657108990966 5097495

[11] Prouty RW, Anderson WH. The forensic science implications of site and temporal influences on postmortem blood-drug concentrations. *J Forensic Sci* 1990;35:243–70. doi: 10.1520/JFS12828J 2329329

[12] Pounder DJ, Owen V, Quigley C. Postmortem changes in blood amitriptyline concentration. *Am J Forensic Med Pathol* 1994;15:224–30. doi: 10.1097/00000433-199409000-00009 7825553

[13] Pounder DJ, Adams E, Fuke C et al. Site to site variability of postmortem drug concentrations in liver and lung. *J Forensic Sci* 1996;41:927–32. doi: 10.1520/JFS14027J 8914282

[14] Shiota H, Nakashima M, Terazono H et al. Postmortem changes in tissue concentrations of triazolam and diazepam in rats. *Leg Med* 2004;6:224–32. doi: 10.1016/j.legalmed.2004.05.006 15363447

[15] Skopp G . Postmortem toxicology. *Forensic Sci Med Pathol* 2010;6:314–25. doi: 10.1007/s12024-010-9150-4 20204545

[16] Donaldson AE, Lamont IL, Appanna V. Biochemistry changes that occur after death: potential markers for determining postmortem interval. *PLoS One* 2013;8:e82011. doi: 10.1371/journal.pone.0082011 PMC3836773 24278469

[17] Butzbach DM . The influence of putrefaction and sample storage on postmortem toxicology results. *Forensic Sci Med Pathol* 2010;6:35–45. doi: 10.1007/s12024-009-9130-8 19946767

[18] Jones GR, Pounder DJ. Site dependence of drug concentrations in postmortem blood—a case study. *J Anal Toxicol* 1987;11:186–90. doi: 10.1093/jat/11.5.186 3682777

[19] Pounder DJ, Jones GR. Postmortem drug redistribution—a toxicological nightmare. *Forensic Sci Int* 1990;45:253–63. doi: 10.1016/0379-0738(90)90182-x 2361648

[20] Bynum ND, Poklis JL, Gaffney-Kraft M et al. Postmortem distribution of tramadol, amitriptyline, and their metabolites in a suicidal overdose. *J Anal Toxicol* 2005;29:401–06. doi: 10.1093/jat/29.5.401 16105270

[21] Øiestad Å, L. M, Karinen R et al. Comparative study of postmortem concentrations of antidepressants in several different matrices. *J Anal Toxicol* 2018;42:446–58. doi: 10.1093/jat/bky030 29762694

[22] Apple FS, Bandt CM. Liver and blood postmortem tricyclic antidepressant concentrations. *Am J Clin Pathol* 1988;89:794–96. doi: 10.1093/ajcp/89.6.794 3369373

[23] Hilberg T, Bugge A, Beylich K-M et al. Diffusion as a mechanism of postmortem drug redistribution: an experimental study in rats. *Int J Legal Med* 1992;105:87–91. doi: 10.1007/BF02340830 1520643

[24] Koren G, Klein J. Postmortem redistribution of morphine in rats. *Ther Drug Monit* 1992;14:461–63. doi: 10.1097/00007691-199212000-00004 1485366

[25] Kudo K, Nagata T, Kimura K et al. Postmortem changes of triazolam concentrations in body tissues. *Nihon Hoigaku Zasshi* 1992;46:293–96. 1460792

[26] Pounder DJ, Davies JI. Zopiclone poisoning: tissue distribution and potential for postmortem diffusion. *Forensic Sci Int* 1994;65:177–83. doi: 10.1016/0379-0738(94)90273-9 8039775

[27] Pounder DJ, Smith DR. Postmortem diffusion of alcohol from the stomach. *Am J Forensic Med Pathol* 1995;16:89–96. doi: 10.1097/00000433-199506000-00001 7572882

[28] Takayasu T, Ohshima T, Tanaka N et al. Experimental studies on postmortem diffusion of ethanol-d6 using rats. *Forensic Sci Int* 1995;76:179–88. doi: 10.1016/0379-0738(95)01820-4 8566920

[29] Pounder DJ, Fuke C, Cox DE et al. Postmortem diffusion of drugs from gastric residue: an experimental study. *Am J Forensic Med Pathol* 1996;17:1–7. doi: 10.1097/00000433-199603000-00001 8838463

[30] Hořínková J, Kozlík P, Křížek T et al. Postmortem redistribution of Alprazolam in rats. *Prague Med Rep* 2020;121:244–53. doi: 10.14712/23362936.2020.21 33270012

[31] Han E, Kim E, Hong H et al. Evaluation of postmortem redistribution phenomena for commonly encountered drugs. *Forensic Sci Int* 2012;219:265–71. doi: 10.1016/j.forsciint.2012.01.016 22284073

[32] Bartschat S, Mercer-Chalmers-Bender K, Beike J et al. Not only smoking is deadly: fatal ingestion of e-juice-a case report. *Int J Legal Med* 2015;129:481–86. doi: 10.1007/s00414-014-1086-x 25239221

[33] Pos Pok PR, Haddouche D, Mauras M et al. Cardiac and peripheral blood similarities in the comparison of nordiazepam and bromazepam blood concentrations. *J Anal Toxicol* 2008;32:782–86. doi: 10.1093/jat/32.9.782 19021936

[34] Moriya F, Hashimoto Y. Redistribution of basic drugs into cardiac blood from surrounding tissues during early-stages postmortem. *J Forensic Sci* 1999;44:10–16. doi: 10.1520/JFS14405J 9987864

[35] Eckes L, Tsokos M, Herre S et al. Postmortem evidence of doxylamine in toxicological analyses. *Sci Justice* 2014;54:61–65. doi: 10.1016/j.scijus.2013.10.005 24438779

[36] Rang HP, Ritter JM, Flower RJ et al. *Rang & Dale’s Pharmacology*. London: Elsevier, 2016.

[37] Luo Q, Zhan W, Boom RM et al. Interactions between acid and proteins under in vitro gastric condition – a theoretical and experimental quantification. *Food Funct* 2018;9:5283–89. doi: 10.1039/C8FO01033A 30250956

[38] Cao Y, Wang M, Yuan Y et al. Arterial blood gas and acid-base balance in patients with pregnancy-induced hypertension syndrome. *Exp Ther Med* 2019;17:349–53. doi: 10.3892/etm.2018.6893 30651802 PMC6307481

[39] Davis PJ Bosenberg A Davidson A et al. Chapter 7 - pharmacology of pediatric anesthesia. In: Davis PJ, Cladis FP, Motoyama EK (eds.), *Smith’s Anesthesia for Infants and Children*. Philadelphia: Mosby, 2011, 179–261.

[40] Hilberg T, Ripel A, Slørdal L et al. The extent of postmortem drug redistribution in a rat model. *J Forensic Sci* 1999;44:956–62. doi: 10.1520/JFS12023J 10486948

[41] Castaing N, Titier K, Canal-Raffin M et al. Postmortem redistribution of two antipsychotic drugs, haloperidol and thioridazine, in the rat. *J Anal Toxicol* 2006;30:419–25. doi: 10.1093/jat/30.7.419 16959133

[42] Saar E, Beyer J, Gerostamoulos D et al. The time-dependant postmortem redistribution of antipsychotic drugs. *Forensic Sci Int* 2012;222:223–27. doi: 10.1016/j.forsciint.2012.05.028 22748972

[43] Breivik H, Frost J, Løkken TN et al. Post mortem tissue distribution of quetiapine in forensic autopsies. *Forensic Sci Int* 2020;315:110413. doi: 10.1016/j.forsciint.2020.110413 32745884

[44] Drummer OH, Gerostamoulos D. Postmortem redistribution of drugs and other factors affecting interpretation: a review. *WIREs Forensic Sci* 2023;5:e1480. doi: 10.1002/wfs2.1480

[45] Koren G, MacLeod SM. Postmortem redistribution of digoxin in rats. *J Forensic Sci* 1985;30:92–96. doi: 10.1520/JFS10968J 3981125

[46] Frommer DA, Kulig KW, Marx JA et al. Tricyclic antidepressant overdose: a review. *JAMA* 1987;257:521–26. doi: 10.1001/jama.1987.03390040137034 3540330

[47] Yamazaki M, Wakasugi C. Postmortem changes in drug-metabolizing enzymes of rat liver microsome. *Forensic Sci Int* 1994;67:155–68. doi: 10.1016/0379-0738(94)90086-8 7959472

[48] Skopp G, Pötsch L, Klingmann A et al. Stability of morphine, morphine-3-glucuronide, and morphine-6-glucuronide in fresh blood and plasma and postmortem blood samples. *J Anal Toxicol* 2001;25:2–7. doi: 10.1093/jat/25.1.2 11215995

[49] Cone EJ, Welch P, Mitchell JM et al. Forensic drug testing for opiates: I. Detection of 6-acetylmorphine in urine as an indicator of recent heroin exposure; drug and assay considerations and detection times. *J Anal Toxicol* 1991;15:1–7. doi: 10.1093/jat/15.1.1 2046334

[50] Jones AW, Holmgren A, Ahlner J. Concentrations of free-morphine in peripheral blood after recent use of heroin in overdose deaths and in apprehended drivers. *Forensic Sci Int* 2012;215:18–24. doi: 10.1016/j.forsciint.2011.01.043 21353406

[51] Maas A, Madea B, Hess C. Confirmation of recent heroin abuse: accepting the challenge. *Drug Test Anal* 2018;10:54–71. doi: 10.1002/dta.2244 28681463

[52] Druid H, Holmgren P, Carlsson B et al. Cytochrome P450 2D6 (CYP2D6) genotyping on postmortem blood as a supplementary tool for interpretation of forensic toxicological results. *Forensic Sci Int* 1999;99:25–34. doi: 10.1016/s0379-0738(98)00169-8 10069020

[53] Butzbach DM, Stockham PC, Kobus HJ et al. Bacterial degradation of risperidone and paliperidone in decomposing blood. *J Forensic Sci* 2013;58:90–100. doi: 10.1111/j.1556-4029.2012.02280.x 22994980

[54] O’Neal CL, Poklis A. Postmortem production of ethanol and factors that influence interpretation: a critical review. *Am J Forensic Med Pathol* 1996;17:8–20. doi: 10.1097/00000433-199603000-00002 8838464

[55] Kugelberg FC, Jones AW. Interpreting results of ethanol analysis in postmortem specimens: a review of the literature. *Forensic Sci Int* 2007;165:10–29. doi: 10.1016/j.forsciint.2006.05.004 16782292

[56] Hanzlick R . Ethanol concentration in decomposing bodies: another look, less concern. *Am J Forensic Med Pathol* 2009;30:88–89. doi: 10.1097/PAF.0b013e3181873814 19237865

[57] Flanagan RJ, Connally G, Evans JM. Analytical toxicology: guidelines for sample collection postmortem. *Toxicol Rev* 2005;24:63–71. doi: 10.2165/00139709-200524010-00005 16042505

[58] Furumiya J, Nishimura H, Nakanishi A et al. Postmortem endogenous ethanol production and diffusion from the lung due to aspiration of wood chip dust in the work place. *Leg Med* 2011;13:210–12. doi: 10.1016/j.legalmed.2011.04.004 21592843

[59] Boumba VA, Economou V, Kourkoumelis N et al. Microbial ethanol production: experimental study and multivariate evaluation. *Forensic Sci Int* 2012;215:189–98. doi: 10.1016/j.forsciint.2011.03.003 21470803

[60] Boumba VA, Kourkoumelis N, Gousia P et al. Modeling microbial ethanol production by E. coli under aerobic/anaerobic conditions: applicability to real postmortem cases and to postmortem blood derived microbial cultures. *Forensic Sci Int* 2013;232:191–98. doi: 10.1016/j.forsciint.2013.07.021 24053880

[61] Roth RH . Formation and regional distribution of gamma-hydroxybutyric acid in mammalian brain. *Biochem Pharmacol* 1970;19:3013–19. doi: 10.1016/0006-2952(70)90087-0 5513038

[62] Beránková K, Mutnanská K, Balíková M. Gamma-hydroxybutyric acid stability and formation in blood and urine. *Forensic Sci Int* 2006;161:158–62. doi: 10.1016/j.forsciint.2006.01.017 16857333

[63] Elliott SP . Further evidence for the presence of GHB in postmortem biological fluid: implications for the interpretation of findings. *J Anal Toxicol* 2004;28:20–26. doi: 10.1093/jat/28.1.20 14987420

[64] Robertson MD, Drummer OH. Postmortem drug metabolism by bacteria. *J Forensic Sci* 1995;40:382–86. doi: 10.1520/JFS13791J 7782744

[65] Batziris HP, McIntyre IM, Drummer OH. The effect of sulfur-metabolising bacteria on sulfur-containing psychotropic drugs. *Int Biodeterior Biodegrad* 1999;44:111–16. doi: 10.1016/S0964-8305(99)00066-9

[66] Butzbach DM, Stockham PC, Kobus HJ et al. Stability of serotonin-selective antidepressants in sterile and decomposing liver tissue. *J Forensic Sci* 2013;58:S117–125. doi: 10.1111/j.1556-4029.2012.02266.x 22931374

[67] Zhou C, Byard RW. Factors and processes causing accelerated decomposition in human cadavers – an overview. *J Forensic Leg Med* 2011;18:6–9. doi: 10.1016/j.jflm.2010.10.003 21216371

[68] Shen M, Shi Y, Xiang P. CYP3A4 and CYP2C19 genetic polymorphisms and zolpidem metabolism in the Chinese Han population: a pilot study. *Forensic Sci Int* 2013;227:77–81. doi: 10.1016/j.forsciint.2012.08.035 22964165

[69] Gasche Y, Daali Y, Fathi M et al. Codeine intoxication associated with ultrarapid CYP2D6 metabolism. *N Engl J Med* 2004;351:2827–31. doi: 10.1056/NEJMoa041888 15625333

[70] Lamba JK, Lin YS, Schuetz EG et al. Genetic contribution to variable human CYP3A-mediated metabolism. *Adv Drug Delivery Rev* 2002;54:1271–94. doi: 10.1016/S0169-409X(02)00066-2 12406645

[71] Dumas EO, Pollack GM. Opioid tolerance development: a pharmacokinetic/pharmacodynamic perspective. *AAPS J* 2008;10:537–51. doi: 10.1208/s12248-008-9056-1 18989788 PMC2628209

[72] Seymour A, Oliver JS, Black M. Drug-related deaths among recently released prisoners in the Strathclyde Region of Scotland. *J Forensic Sci* 2000;45:649–54. doi: 10.1520/JFS14741J 10855971

[73] Hilberg T, Rogde S, Mørland J. Postmortem drug redistribution—human cases related to results in experimental animals. *J Forensic Sci* 1999;44:3–9. doi: 10.1520/JFS14404J 9987863

[74] Byard RW, Butzbach DM. Issues in the interpretation of postmortem toxicology. *Forensic Sci Med Pathol* 2012;8:205–07. doi: 10.1007/s12024-011-9278-x 21918893

[75] Tiemensma M, Rutherford JD, Scott T et al. Emergence of Cumyl-PEGACLONE-related fatalities in the Northern Territory of Australia. *Forensic Sci Med Pathol* 2021;17:3–9. doi: 10.1007/s12024-020-00334-0 33185835

[76] deJong JL, Lee J, Grande A et al. Positional asphyxia in opioid-related deaths: is it being overlooked? *J Forensic Sci* 2020;65:2008–12. doi: 10.1111/1556-4029.14524 32745253

[77] Ruttenber AJ, Luke JL. Heroin-related deaths: new epidemiologic insights. *Science* 1984;226:14–20. doi: 10.1126/science.6474188 6474188

[78] Fugelstad A, Ahlner J, Brandt L et al. Use of morphine and 6-monoacetylmorphine in blood for the evaluation of possible risk factors for sudden death in 192 heroin users. *Addiction* 2003;98:463–70. doi: 10.1046/j.1360-0443.2003.00330.x 12653816

[79] Rees KA, Pounder DJ, Osselton MD. Distribution of opiates in femoral blood and vitreous humour in heroin/morphine-related deaths. *Forensic Sci Int* 2013;226:152–59. doi: 10.1016/j.forsciint.2013.01.002 23357228

[80] Dalpe-Scott M, Degouffe M, Garbutt D et al. A comparison of drug concentrations in postmortem cardiac and peripheral blood in 320 cases. *Can Soc Forensic Sci J* 1995;28:113–21. doi: 10.1080/00085030.1995.10757474

[81] Apple FS . A better understanding of the interpretation of postmortem blood drug concentrations. *J Anal Toxicol* 2011;35:381–83. doi: 10.1093/anatox/35.6.381 21740697

[82] Logan BK, Friel PN, Case GA. Analysis of Sertraline (Zoloft^®^) and its major metabolite in postmortem specimens by gas and liquid chromatography. *J Anal Toxicol* 1994;18:139–42. doi: 10.1093/jat/18.3.139 8065122

[83] Rodda KE, Drummer OH. The redistribution of selected psychiatric drugs in postmortem cases. *Forensic Sci Int* 2006;164:235–39. doi: 10.1016/j.forsciint.2006.02.013 16554130

[84] Levine B, Jenkins AJ, Queen M et al. Distribution of Venlafaxine in three postmortem cases. *J Anal Toxicol* 1996;20:502–05. doi: 10.1093/jat/20.6.502 8889688

[85] Parsons AT, Anthony RM, Meeker JE. Two fatal cases of venlafaxine poisoning. *J Anal Toxicol* 1996;20:266–68. doi: 10.1093/jat/20.4.266 8835666

[86] Jaffe PD, Batziris HP, van der Hoeven P et al. A study involving venlafaxine overdoses: comparison of fatal and therapeutic concentrations in postmortem specimens. *J Forensic Sci* 1999;44:193–96. doi: 10.1520/JFS14434J 9987886

[87] Chu M, Rago MD, Mantinieks D et al. Time-dependent changes in THC concentrations in deceased persons. *J Anal Toxicol* 2020;45:1–7. doi: 10.1093/jat/bkaa052 32435813

[88] Kacinko SL, Isenschmid DS, Logan BK. Are postmortem cannabinoid concentrations forensically reliable? *Am J Forensic Med Pathol* 2024;45:92–93. doi: 10.1097/paf.0000000000000887 37801547

[89] Hilberg T, Ripel Å, Slørdal L et al. Postmortem amitriptyline pharmacokinetics in pigs after oral and intravenous routes of administration. *J Forensic Sci* 1998;43:380–87. doi: 10.1520/JFS16151J 9544547

[90] Keller T, Schneider A, Tutsch-Bauer E. GC/MS determination of zolpidem in postmortem specimens in a voluntary intoxication. *Forensic Sci Int* 1999;106:103–08. doi: 10.1016/S0379-0738(99)00185-1 10664896

[91] Vermeulen T . Distribution of paroxetine in three postmortem cases. *J Anal Toxicol* 1998;22:541–44. doi: 10.1093/jat/22.6.541 9788532

[92] Logan BK, Smirnow D, Gullberg RG. Lack of predictable site-dependent differences and time-dependent changes in postmortem concentrations of cocaine, benzoylecgonine, and cocaethylene in humans. *J Anal Toxicol* 1997;21:23–31. doi: 10.1093/jat/21.1.23 9013288

[93] Anderson DT, Muto JJ. Duragesic transdermal patch: postmortem tissue distribution of fentanyl in 25 cases. *J Anal Toxicol* 2000;24:627–34. doi: 10.1093/jat/24.7.627 11043670

[94] Juebner M, Fietzke M, Beike J et al. Assisted suicide by fentanyl intoxication due to excessive transdermal application. *Int J Legal Med* 2014;128:949–56. doi: 10.1007/s00414-014-0982-4 24577713

[95] Prodduturi S, Sadrieh N, Wokovich AM et al. Transdermal delivery of fentanyl from matrix and reservoir systems: effect of heat and compromised skin. *J Pharmaceut Sci* 2010;99:2357–66. doi: 10.1002/jps.22004 19967778

[96] Lane ME . The transdermal delivery of fentanyl. *Eur J Pharm Biopharm* 2013;84:449–55. doi: 10.1016/j.ejpb.2013.01.018 23419814

[97] Levine BS Jufer RA . Drugs-of-abuse testing in vitreous humor. In: Jenkins AJ, Caplan YH (eds.), *Drug Testing in Alternate Biological Specimens*. Totowa, NJ: Humana Press, 2008, 117–30.

[98] Apple FS . Postmortem tricyclic antidepressant concentrations: assessing cause of death using parent drug to metabolite ratio. *J Anal Toxicol* 1989;13:197–98. doi: 10.1093/jat/13.4.197 2779168

[99] Viinamäki J, Ojanperä I. Concurrent estimation of metabolite concentrations along with parent drug quantification in postmortem blood. *Forensic Sci Int* 2016;267:110–14. doi: 10.1016/j.forsciint.2016.08.031 27597253

[100] Garriott JC, Sturner WQ. Morphine concentrations and survival periods in acute heroin fatalities. *N Engl J Med* 1973;289:1276–78. doi: 10.1056/nejm197312132892404 4749546

[101] Staub C, Jeanmonod R, Fryc O. Morphine in postmortem blood: its importance for the diagnosis of deaths associated with opiate addiction. *Int J Legal Med* 1990;104:39–42. doi: 10.1007/bf01816482 11453091

[102] Burt MJ, Kloss J, Apple FS. Postmortem blood free and total morphine concentrations in medical examiner cases. *J Forensic Sci* 2001;46:1138–42. doi: 10.1520/JFS15112J 11569556

[103] Avella J, Katz M, Lehrer M. Assessing free and total morphine following heroin overdose when complicated by the presence of toxic amitriptyline levels. *J Anal Toxicol* 2007;31:540–42. doi: 10.1093/jat/31.8.540 17988471

[104] Spiehler V, Brown R. Unconjugated morphine in blood by radioimmunoassay and gas chromatography/mass spectrometry. *J Forensic Sci* 1987;32:906–16. doi: 10.1520/JFS12402J 3612075

[105] Goldberger BA, Cone EJ, Grant TM et al. Disposition of heroin and its metabolites in heroin-related deaths. *J Anal Toxicol* 1994;18:22–28. doi: 10.1093/jat/18.1.22 8127080

[106] Skopp G, Lutz R, Ganssmann B et al. Postmortem distribution pattern of morphine and morphine glucuronides in heroin overdose. *Int J Legal Med* 1996;109:118–24. doi: 10.1007/bf01369670 8956984

[107] Faura CC, Collins SL, Moore AR et al. Systematic review of factors affecting the ratios of morphine and its major metabolites. *Pain* 1998;74:43–53. doi: 10.1016/s0304-3959(97)00142-5 9514559

[108] Moriya F, Hashimoto Y. Distribution of free and conjugated morphine in body fluids and tissues in a fatal heroin overdose: is conjugated morphine stable in postmortem specimens? *J Forensic Sci* 1997;42:736–40. doi: 10.1520/JFS14195J 9243843

[109] Langford AM, Pounder DJ. Possible markers for postmortem drug redistribution. *J Forensic Sci* 1997;42:88–92. doi: 10.1520/JFS14072J 8988578

[110] Brockbals L, Staeheli SN, Kraemer T et al. Postmortem metabolomics: correlating time-dependent concentration changes of xenobiotic and endogenous compounds. *Drug Test Anal* 2020;12:1171–82. doi: 10.1002/dta.2814 32372514

[111] Brockbals L, Wartmann Y, Mantinieks D et al. Postmortem metabolomics: strategies to assess time-dependent postmortem changes of diazepam, nordiazepam, morphine, codeine, mirtazapine and citalopram. *Metab* 2021;11:643. doi: 10.3390/metabo11090643 PMC8466227 34564459

[112] Stead AH, Moffat AC. A collection of therapeutic, toxic and fatal blood drug concentrations in man. *Hum Toxicol* 1983;2:437–64. doi: 10.1177/096032718300200301 6885090

[113] Meyer FP . Indicative therapeutic and toxic drug concentrations in plasma: a tabulation. *Int J Clin Pharmacol Ther* 1994;32:71–81. 8004362

[114] Repetto MR, Repetto M. Therapeutic, toxic, and lethal concentrations in human fluids of 90 drugs affecting the cardiovascular and hematopoietic systems. *J Toxicol Clin Toxicol* 1997;35:345–51. doi: 10.3109/15563659709043365 9204093

[115] Schulz M, Schmoldt A. Therapeutic and toxic blood concentrations of more than 500 drugs. *Die Pharmazie* 1997;52:895–911. 9442555

[116] Repetto MR, Repetto M. Therapeutic, toxic, and lethal concentrations of 73 drugs affecting respiratory system in human fluids. *J Toxicol Clin Toxicol* 1998;36:287–93. doi: 10.3109/15563659809028023 9711193

[117] Regenthal R, Krueger M, Koeppel C et al. Drug levels: therapeutic and toxic serum/plasma concentrations of common drugs. *J Clin Monit Comput* 1999;15:529–44. doi: 10.1023/a:1009935116877 12578052

[118] Schulz M, Schmoldt A. Therapeutic and toxic blood concentrations of more than 800 drugs and other xenobiotics. *Die Pharmazie* 2003;58:447–74. 12889529

[119] Schulz M, Iwersen-Bergmann S, Andresen H et al. Therapeutic and toxic blood concentrations of nearly 1,000 drugs and other xenobiotics. *Critical Care* 2012;16:R136. doi: 10.1186/cc11441 PMC3580721 22835221

[120] Launiainen T, Ojanperä I. Drug concentrations in postmortem femoral blood compared with therapeutic concentrations in plasma. *Drug Test Anal* 2014;6:308–16. doi: 10.1002/dta.1507 23881890 PMC4237191

[121] Jönsson AK, Söderberg C, Espnes KA et al. Sedative and hypnotic drugs—fatal and non-fatal reference blood concentrations. *Forensic Sci Int* 2014;236:138–45. doi: 10.1016/j.forsciint.2014.01.005 24529785

[122] Ketola RA, Ojanperä I. Summary statistics for drug concentrations in postmortem femoral blood representing all causes of death. *Drug Test Anal* 2019;11:1326–37. doi: 10.1002/dta.2655 31128576

[123] Kriikku P, Ojanperä I. Fatal concentrations of antidepressant and antipsychotic drugs in postmortem femoral blood. *J Anal Toxicol* 2023;47:615–22. doi: 10.1093/jat/bkad044 37440364 PMC10503646

[124] Maskell PD, Elliott S, Desharnais B et al. A model of evaluative opinion to encourage greater transparency and justification of interpretation in postmortem forensic toxicology. *J Anal Toxicol* 2023;47:563–73. doi: 10.1093/jat/bkad055 37566485 PMC10503647

[125] Angerer V, Jacobi S, Franz F et al. Three fatalities associated with the synthetic cannabinoids 5F-ADB, 5F-PB-22, and AB-CHMINACA. *Forensic Sci Int* 2017;281:e9–e15. doi: 10.1016/j.forsciint.2017.10.042 29133010

